# Reduced regional cerebral oxygen saturation increases risk for emergence delirium in pediatric patients

**DOI:** 10.3389/fped.2023.1117455

**Published:** 2023-06-08

**Authors:** Lijing Li, Zhengzheng Gao, Jianmin Zhang, Fuzhou Zhang, Fang Wang, Xiaoxue Wang, Gan Li

**Affiliations:** Department of Anesthesiology, Beijing Children’s Hospital, Capital Medical University, National Center for Children’s Health, Beijing, China

**Keywords:** emergence delirium, regional cerebral oxygen saturation, general anesthesia, children, desaturation

## Abstract

**Objectives:**

To assess whether decreased regional cerebral oxygen saturation (rScO_2_) is associated with the emergence delirium (ED) following general anesthesia in the pediatric population.

**Methods:**

A retrospective observational cohort study was conducted on 113 children (ASA I–III) aged 2–14 years who underwent selective surgery under general anesthesia between 2022-01 and 2022-04. Intraoperatively, the rScO_2_ was monitored using a cerebral oximeter. The Pediatric Anesthesia Emergence Delirium (PAED) score was used to evaluate the patients for ED.

**Results:**

The incidence of ED was 31%. Low rScO_2_ was reported in 41.6% of patients, who had a higher incidence of ED (*P* < 0.001) than those who did not experience desaturation. Logistic regression analysis revealed that decreased rScO_2_ was significantly associated with incident ED events [odds ratio (OR), 10.77; 95% confidence interval, 3.31–35.05]. Children under 3 years of age had a higher incidence of ED after rScO_2_ desaturation during anesthesia compared to older children (OR, 14.17 vs. 4.64).

**Conclusion:**

Intraoperative rScO_2_ desaturation significantly increased the incidence of ED following general anesthesia. Monitoring should be enhanced to improve the oxygen balance in vital organs to improve the quality and safety of anesthesia.

## Introduction

The occurrence of emergence delirium (ED) in children is related to emergence quality and medical care cost, and its prevalence varies widely in different studies, ranging up to 80% (1–3). ED is considered a type of behavioral disorder and a neurological complication that develops after general anesthesia in children and can involve hypoactive (i.e., lethargy and inattentiveness) or hyperactive (i.e., agitation and restlessness) signs, or a mixture. The most common ED risk factors are the presence of endotracheal intubation, preschool age, volatile anesthetics, ophthalmologic and otolaryngology surgical procedures, history of behavioral problems, negative behavior on induction, preoperative anxiety level, and postoperative pain (4). Studies on delirium among surgical patients have found that this brain organ dysfunction is independently associated with prolonged cognitive impairment, extended hospital stays, increased cost of care, and increased mortality (5–7). Previous studies have suggested that reduced regional cerebral oxygen saturation (rScO_2_) during surgery may also be clinically relevant to cognitive dysfunction (8, 9), and may contribute to the development of ED. Near-infrared spectroscopy (NIRS) allows real-time, noninvasive monitoring of rScO_2_ (10). Therefore, NIRS may help identify pediatric patients at risk for ED and alleviate brain desaturation during general anesthesia.

Intraoperative decreases in basal rScO_2_ values of 20% or more are harmful and are associated with postoperative cognitive dysfunction in adults (11). However, the level of brain function impairment due to intraoperative rScO_2_ reduction is unclear. According to studies, severe rScO_2_ reduction during non-cardiac surgery is rare compared with mid- or low-level rScO_2_ reduction in the pediatric population (12, 13). Previous studies demonstrated that even a decrease in rScO_2_ of less than 10% from baseline values might reflect high intraoperative bleeding or postoperative behavioral changes in children (12, 14). This is attributable to how the brains of children are immature, have different tolerance, and regulate hypoxia differently compared to adults. Thus, we defined rScO_2_ >10% below baseline values as regional cerebral oxygen desaturation.

The primary objective of this study was to assess whether a decline in rScO_2_ of >10% from baseline levels was correlated with an elevated incidence of ED in pediatric patients undergoing general anesthesia.

## Methods

### Study design

This retrospective observational study was performed at Beijing Children's Hospital, China. The use of fully anonymized cohort data for research purposes was approved by the Ethics Committee of Beijing Children's Hospital, Capital Medical University, National Center for Children's Health (2021-E-114-Y), without the need for informed consent. All data were anonymized for extraction and analysis.

### Population

We identified a cohort of children aged 2–14 years with rScO_2_ monitoring using NIRS during surgery under total intravenous general anesthesia between 2022 and 01-01 and 2022-04-30. Children with autism, developmental delay, cognitive impairment, or neurological or neuromuscular diseases were excluded.

### Anesthesia management

Anesthesia was induced using propofol 2–3 mg·kg^−1^, sufentanil 0.3–0.5 mcg·kg^−1^, and cisatracurium 0.1 mg·kg^−1^ to facilitate endotracheal intubation. After tracheal intubation, an anesthesia machine was connected to control the breathing. Anesthesia was maintained using propofol (8–10 mg·kg^−1^·h^−1^) and remifentanil (0.3–0.4 mcg·kg^−1^·min^−1^), with the dose adjusted to the analgesic requirements (systolic blood pressure maintained within 20% of baseline values). Ibuprofen (10 mg·kg^−1^ i.v.) was administered intraoperatively for postoperative analgesia, and fentanyl was used as required during emergence. Mechanical ventilation was adjusted to maintain an end-tidal CO_2_ between 35 and 45 mmHg. Body temperature was monitored using a nasopharyngeal thermometer and was maintained between 36.5°C and 37.5°C using a warm air blanket.

### Assessment of ED and preoperative anxiety status

The Pediatric Anesthesia Emergence Delirium (PAED) score was used to assess whether a child experienced delirium after general anesthesia (Supplementary Table S1). While the patients were undergoing recovery in the PACU, a measurement based on the PAED scale scores within 15 min following extubation. The restlessness and agitation appeared within 15 min, an assessment of the agitation and pain degree was started immediately. The onset of ED was defined as the first evaluation of each patient with a PAED score ≥10. The Pediatric Anesthesia Behavior (PAB) score was used to evaluate children's behavior and mood before the induction of anesthesia (Supplementary Table S2).

### Data collection

Data were routinely collected using a standardized electronic anesthesia system (Docare, MedicalSystem Company). Information collected included demographic data (i.e., age, sex, and weight), PAB and PAED scores, rScO_2_ level, type of surgery, intraoperative hemorrhage, and anesthesia duration. Cerebral NIRS was performed using the FORE-SIGHT ELITE Cerebral Oxygen Saturation Monitor (NIRS, CAS Medical Systems Inc., Branford, CT, USA) with the medium bihemispheric sensors. When used with medium sensors, the module is indicated for use on pediatric subjects ≥3 kg. The sensor was placed bilaterally on the patient's forehead prior to the induction of anesthesia in operating room, and the rScO_2_ value was considered the baseline, for children who couldn't cooperate, we would use toys or animations to distract them and gain enough time to obtain the basal values; rScO_2_ values of the right and left frontal monitors were recorded simultaneously. The pooled value of rScO_2_ (mean value of the left and right sides) was used for analysis. Cerebral desaturation was defined as a decrease in rScO_2_ of ≥10% from the baseline for at least 3 min. Depending on the degree of decline of rScO_2_ compared to the baseline, we divided it into three levels and two groups: normal rScO_2_ group: decrease <10% (i.e., no desaturation); low rScO_2_ group: decrease of 10%–20% or >20%.

### Statistical analyses

Histograms and the Kolmogorov–Smirnov test were used to assess normality. Continuous variables were expressed as the mean ± standard deviation or median (interquartile interval), as appropriate. To assess differences between the two groups, the *t*-test was used for normally distributed continuous variables, whereas the Wilcoxon rank-sum test was used for non-normally distributed continuous variables. For categorical variables, the *χ*^2^ test and Fisher's exact test were used. We further characterized associations between ED and rScO_2_ levels by logistic regression analyses adjusted for age, sex, weight, surgery type, PAB and PAED scores, hemorrhage, and anesthesia duration. We calculated the odds ratios (ORs) with 95% confidence intervals (CIs) for the risk of ED, progressively adjusted for the above variables. Statistical analyses were performed using the International Business Machines Statistical Package for the Social Sciences (SPSS) Statistics version 21.0 (SPSS Inc., Chicago, IL, USA) and GraphPad Prism 9.1 (GraphPad Software Company, San Diego, CA, USA). We selected a significance threshold of *P* < 0.05 for comparisons between groups.

## Results

The enrollment data and patient demographic characteristics of the 113 patients included in this study are summarized in Table 1. There were no statistically significant differences in age, sex, weight, and PAB scores between the two groups. The incidence of ED was 31.0% (35/113). Low rScO_2_ was reported in 41.6% (47/113) of patients who had higher PAED scores (*P* < 0.001) and with a higher incidence of ED (*P* < 0.001) than patients who did not experience desaturation during general anesthesia. In addition, patients in the low rScO_2_ group had greater bleeding and longer duration of anesthesia than those in the normal rScO_2_ group.

**Table 1:** Characteristics of participants with or without low rScO_2_.

	Total *n* = 113	Normal rScO_2_ *n* = 66	Low rScO_2_ *n* = 47	*P* value
Age (years)	5.0 (3.0–9.0)	5.0 (3.0–8.0)	5.8 (3.0–9.5)	0.365
Sex				0.434
Males	53 (46.9)	33 (50.0)	20 (42.6)	
Females	60 (53.1)	33 (50.0)	27 (57.4)	
Weight (kg)	20.0 (15.5–28.2)	20.0 (14.5–28.0)	21.6 (15.6–32.0)	0.423
PAB score	1.0 (1.0–2.0)	1.0 (1.0–2.0)	2.0 (1.0–2.0)	0.435
PAED score	6.0 (3.0–10.0)	5.0 (2.0–8.0)	10.0 (3.0–10.0)	<0.001*
Emergence delirium	35 (31.0)	10 (15.2)	25 (53.2)	<0.001*
Surgery types				<0.001*
Scoliosis	39 (34.5)	12 (18.2)	27 (57.4)	
Fracture	17 (15.1)	9 (13.6)	8 (17.0)	
Abdominal tumor	20 (17.7)	15 (22.7)	5 (10.6)	
Hypospadias	7 (6.2)	6 (9.1)	1 (2.1)	
Hydronephrosis	19 (16.8)	16 (24.2)	3 (6.4)	
Biliary tract	11 (9.7)	8 (12.1)	3 (6.4)	
Hemorrhage (ml)	15.0 (5.0–200.0)	5.0 (3.0–50.0)	140.0 (10.0–420.0)	<0.001*
Anesthesia duration	195.0 (130.0–267.5)	155.0 (118.8–252.5)	210.0 (170.0–275.0)	0.014*

Data are presented as median (interquartile range), or number of cases. PAB, pediatric anesthesia behavior; PAED, pediatric anesthesia emergence delirium score; rScO_2_, regional cerebral oxygen saturation.

* *P* < 0.05.

**Figure 1:**
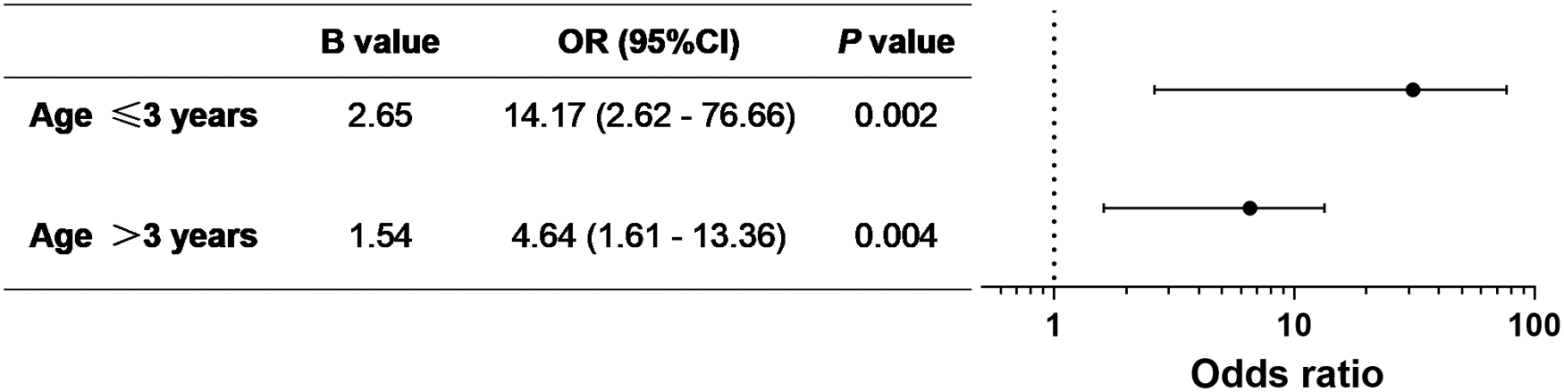
Logistic regression analysis of the association between rScO_2_ desaturation and ED in different age groups.

After adjusting for age, sex, and weight, the OR for ED was 7.88 (95% CI, 3.07–20.21) in patients with rScO_2_ desaturation compared with those with normal rScO_2_ during general anesthesia (Table 2). After progressive adjustment for various independent variables, decreased rScO_2_ remained significantly associated with incident ED events (OR, 10.77; 95% CI, 3.31–35.05). Logistic regression analyses with different levels of decreased rScO_2_ showed that patients with rScO_2_ decline of >20% had approximately 20 times higher odds of developing ED than patients with no rScO_2_ desaturation. This value was approximately seven-fold higher for rScO_2_ decline between 10% and 20%. Subgroup analysis by age (Figure 1) revealed a higher incidence of ED during the emergence period after experiencing rScO_2_ desaturation under anesthesia when patient was younger than 3 years old than in older children (OR, 14.17 vs. 4.64).

**Table 2:** Association of decreased rScO_2_ with incident ED events.

	B value	OR (95% CI)	*P* value
Not adjusted	1.85	6.36 (2.63–15.40)	<0.001*
Adjusted for age, sex, weight	2.06	7.88 (3.07–20.21)	<0.001*
Plus the PAB score	2.07	7.96 (0.59–1.01)	<0.001*
Plus surgery types	2.37	10.68 (3.32–34.37)	<0.001*
Plus hemorrhage	2.37	10.74 (3.31–34.90)	<0.001*
Plus anesthesia duration	2.38	10.77 (3.31–35.05)	<0.001*
Different levels of decreased rScO_2_			
Decrease <10%	Reference	Reference	Reference
Decrease 10%–20%	2.01	7.40 (2.29–23.88)	0.001*
Decrease >20%	3.04	20.84 (2.46–176.53)	0.005*

Data are odds ratio (95% CI). Logistic regression analyses with different levels of decreased rScO_2_ adjusted for all the above-mentioned independent variables. rScO_2_, regional cerebral oxygen saturation; ED, emergence delirium; PAB, pediatric anesthesia behavior.

* *P* < 0.05.

## Discussion

This study found that among children who received general anesthesia, a decrease in rScO_2_ of more than 10% from baseline was associated with ED. There is no standardized definition for pathological brain region desaturation (15, 16), specifically among children. The definition of hypoxia is based on a comparison of rScO_2_ with its own baseline. Children exhibit wide variations in baseline rScO_2_ because of comorbidities and their immature brains are more vulnerable to anesthetics (17). Our study was conducted on a heterogeneous population of children undergoing various operations, such as urologic, gastrointestinal, and orthopedic surgeries.

Cerebral perfusion index, systemic oxygenation, and cerebral metabolism, which may be influenced by anesthesia, can affect rScO_2_ values (18, 19). In addition, oxygen extraction from cerebral neurons affects rScO_2_ values; impaired extraction manifests as normal or increased rScO_2_ values. Our patients were less likely to suffer from impaired cerebral oxygen extraction because their underlying physical condition was generally good, while they maintained sufficient oxygen saturation (SpO_2_ > 98%) and adequate body temperature and hemodynamics during general anesthesia.

Cerebral NIRS measures cerebral oxygen saturation in the region under the forehead stickers. However, in children, this regional cerebral oxygen saturation may reflect the balance between the consumption and supply of oxygen of not only in a local area under the forehead but also in most of the brain due to a more immature self-regulating cerebral system (20, 21). This may explain why, in our study, a reduction in rScO_2_ of >10% of baseline was associated with increased odds of ED (OR 10.77), even if the reduction was less than the threshold of 20% commonly used in adults. This was consistent with Gómez-Pesquera et al. (14). The analysis of different levels of decreased rScO_2_ showed that pediatric patients whose rScO_2_ desaturation exceeded 20% of the baseline under general anesthesia had a higher risk of ED (OR 20.84 vs. 7.40). Of all patients in this study, only seven had rScO_2_ desaturation >20% below baseline; of these, five experienced ED. The more severe rScO_2_ desaturation, the higher incidence of ED. Therefore, when using intraoperative rScO_2_ monitoring in pediatric patients, the threshold criteria for rScO_2_ desaturation should be decreased, and the anesthesiologist must be alert when desaturation decreases by >10%.

Younger pediatric patients with immature brains might be more vulnerable to anesthetic effects (22–24). Thus, we stratified the data by age to determine differences in the impact of rScO_2_ desaturation on ED at different ages (Figure 1). In our cohort, rScO_2_ desaturation could lead to ED regardless of the patient's age; i.e., rScO_2_ desaturation was an independent risk factor for ED. Children younger than 3 years who experienced rScO_2_ desaturation during general anesthesia were at greater risk of ED than those older than 3 years. The younger the age, the more severe the effect of rScO_2_ desaturation.

This study had limitations. First, rScO_2_ monitoring was not a routine clinical practice at our institute. The number of cases included in the analysis was small, especially when a stratified analysis was performed. Therefore, the OR value for rScO_2_ desaturation ≥20% had a wider CI. Second, our study focused on the implications of decreased rScO_2_, and we did not analyze increases in rScO_2_ from the baseline, which are also related to cognitive deterioration. Third, we did not evaluate other factors that may influence the development of ED, such as intraoperative and postoperative pain; a future study on risk factor analysis might choose to include these factors.

In conclusion, a >10% decrease in rScO_2_ from baseline was associated with ED among children who received general anesthesia. Patients under 3 years old were more likely to develop ED after experiencing rScO_2_ desaturation under general anesthesia. Intraoperative monitoring should be strengthened to maintain the oxygen supply and demand balance of vital organs, reduce complications, improve the quality of anesthesia, and ensure patient safety.

## Data Availability

The original contributions presented in the study are included in the article/**Supplementary Material**, further inquiries can be directed to the corresponding author.
