# Supplementary material for: Reduced regional cerebral oxygen saturation increases risk for emergence delirium in pediatric patients
Source: Front Pediatr. 2023 Jun 8;11:1117455. doi: 10.3389/fped.2023.1117455 (PMC10285695; doi:10.3389/fped.2023.1117455)
Supplement: Supplementary file 1 [file Datasheet1.pdf]

frontiers

Supplementary Material

Reduced regional cerebral oxygen saturation increases risk for emergence delirium in pediatric patients

Lijing Li[1], Zhengzheng Gao[1], Jianmin Zhang[1*], Fuzhou Zhang[1], Fang Wang[1], Xiaoxue Wang[1], and Gan Li[1]

[1]Department of Anesthesiology, Beijing Children's Hospital, Capital Medical University, National Center for Children's Health, Beijing, China

[*]Correspondence:

Jianmin Zhang

zhangjianmin@bch.com.cn

1 Supplementary Tables

Supplemental Table 1. The Pediatric Anesthesia Emergence Delirium Scale

1. The child makes eye contact with the caregiver.
2. The child's actions are purposeful.
3. The child is aware of his/her surroundings.
4. The child is restless.
5. The child is inconsolable.

Items 1, 2, and 3 are reversed scored as follows: 4=not at all, 3=just a little, 2=quite a bit, 1=very much, 0=extremely. Items 4 and 5 are scored as follows: 0=not at all, 1=just a little, 2=quite a bit, 3=very much, 4=extremely. The scores of each item were summed to obtain a total Pediatric Anesthesia Emergence Delirium (PAED) scale score. The degree of emergence delirium increased directly with the total score.

Supplemental Table 2. The Pediatric Anesthesia Behavior Score

Score		Description of behaviour
1	Happy	Calm and controlled. Compliant with induction
2	Sad	Tearful and/or withdrawn but compliant with induction
3	Mad	Loud vocal resistance (screaming or shouting) AND/OR Physical resistance to induction requiring physical restraint by staff and/or parents
